# Mechanistic Coupling of a Novel *in silico* Cotyledon Perfusion Model and a Physiologically Based Pharmacokinetic Model to Predict Fetal Acetaminophen Pharmacokinetics at Delivery

**DOI:** 10.3389/fped.2021.733520

**Published:** 2021-09-23

**Authors:** Paola Mian, Bridget Nolan, John N. van den Anker, Kristel van Calsteren, Karel Allegaert, Nisha Lakhi, André Dallmann

**Affiliations:** ^1^Department of Clinical Pharmacy, Medisch Spectrum Twente, Enschede, Netherlands; ^2^Department of Obstetrics and Gynecology, Richmond University Medical Center, Staten Island, NY, United States; ^3^Department of Obstetrics and Gynecology, New York Medical College, Valhalla, NY, United States; ^4^Division of Clinical Pharmacology, Children's National Hospital, Washington, DC, United States; ^5^Department of Pediatric Pharmacology and Pharmacometrics, University Children's Hospital Basel, Basel, Switzerland; ^6^Department of Development and Regeneration, KU Leuven, Leuven, Belgium; ^7^Department of Gynecology and Obstetrics, UZ Gasthuisberg, Leuven, Belgium; ^8^Department of Pharmaceutical and Pharmacological Sciences, KU Leuven, Leuven, Belgium; ^9^Department of Hospital Pharmacy, Erasmus Medical Center Rotterdam, Rotterdam, Netherlands; ^10^Pharmacometrics/Modeling and Simulation, Research and Development, Pharmaceuticals, Bayer AG, Leverkusen, Germany

**Keywords:** acetaminophen, *ex vivo* cotyledon perfusion, physiologically-based pharmacokinetics, placental transfer, maternal-fetal, pregnancy

## Abstract

Little is known about placental drug transfer and fetal pharmacokinetics despite increasing drug use in pregnant women. While physiologically based pharmacokinetic (PBPK) models can help in some cases to shed light on this knowledge gap, adequate parameterization of placental drug transfer remains challenging. A novel *in silico* model with seven compartments representing the *ex vivo* cotyledon perfusion assay was developed and used to describe placental transfer and fetal pharmacokinetics of acetaminophen. Unknown parameters were optimized using observed data. Thereafter, values of relevant model parameters were copied to a maternal-fetal PBPK model and acetaminophen pharmacokinetics were predicted at delivery after oral administration of 1,000 mg. Predictions in the umbilical vein were evaluated with data from two clinical studies. Simulations from the *in silico* cotyledon perfusion model indicated that acetaminophen accumulates in the trophoblasts; simulated steady state concentrations in the trophoblasts were 4.31-fold higher than those in the perfusate. The whole-body PBPK model predicted umbilical vein concentrations with a mean prediction error of 24.7%. Of the 62 concentration values reported in the clinical studies, 50 values (81%) were predicted within a 2-fold error range. In conclusion, this study presents a novel *in silico* cotyledon perfusion model that is structurally congruent with the placenta implemented in our maternal-fetal PBPK model. This allows transferring parameters from the former model into our PBPK model for mechanistically exploring whole-body pharmacokinetics and concentration-effect relationships in the placental tissue. Further studies should investigate acetaminophen accumulation and metabolism in the placenta as the former might potentially affect placental prostaglandin synthesis and subsequent fetal exposure.

## Introduction

Despite frequent and increasing drug use in pregnant women (1, 2), little is known about placental drug transfer and pharmacokinetics in the fetus. To address this knowledge gap, numerous physiologically based pharmacokinetic (PBPK) models for pregnant women were developed over the past years and used to simulate fetal pharmacokinetics (3, 4). Yet, adequate parameterization of placental drug transfer in these models remains challenging. While some models relied on various *in vitro* information, such as the drug's physicochemical properties or permeability across Caco-2 cell membranes, to estimate placental drug transfer (5–8), other models integrated kinetic data obtained from the *ex vivo* cotyledon perfusion assay (9–14) or fitted the placental permeability to clinical data (15).

The kinetic *in silico* models representing the *ex vivo* cotyledon perfusion system typically consist of few compartments and lump various tissue portions of the cotyledon, e.g., intravillous vascular, interstitial, and intracellular space, in a single compartment. Although in general these models appear to scale well with the placental drug transfer kinetics simulated in whole-body models, the relatively simple structure prevents a more mechanistic understanding of the transfer kinetics. For example, drug accumulation in the trophoblasts of the cotyledon—which may lengthen fetal drug exposure *in vivo* (16)—cannot be described by these models. Along the same line, the understanding of placental concentration-time profiles enables modeling concentration-effects profiles in the placental tissue, as the placenta is not only a ‘transfer' organ but an ‘active' organ with endocrine and metabolic functions. Hence, tissue-specific pharmacology of a given drug within the placenta and potential interactions with its endocrine synthesis and secretion of hormones (e.g., prostaglandins) may also affect fetal development and pregnancy outcome (e.g., preterm induction of labor).

**Figure 1:**
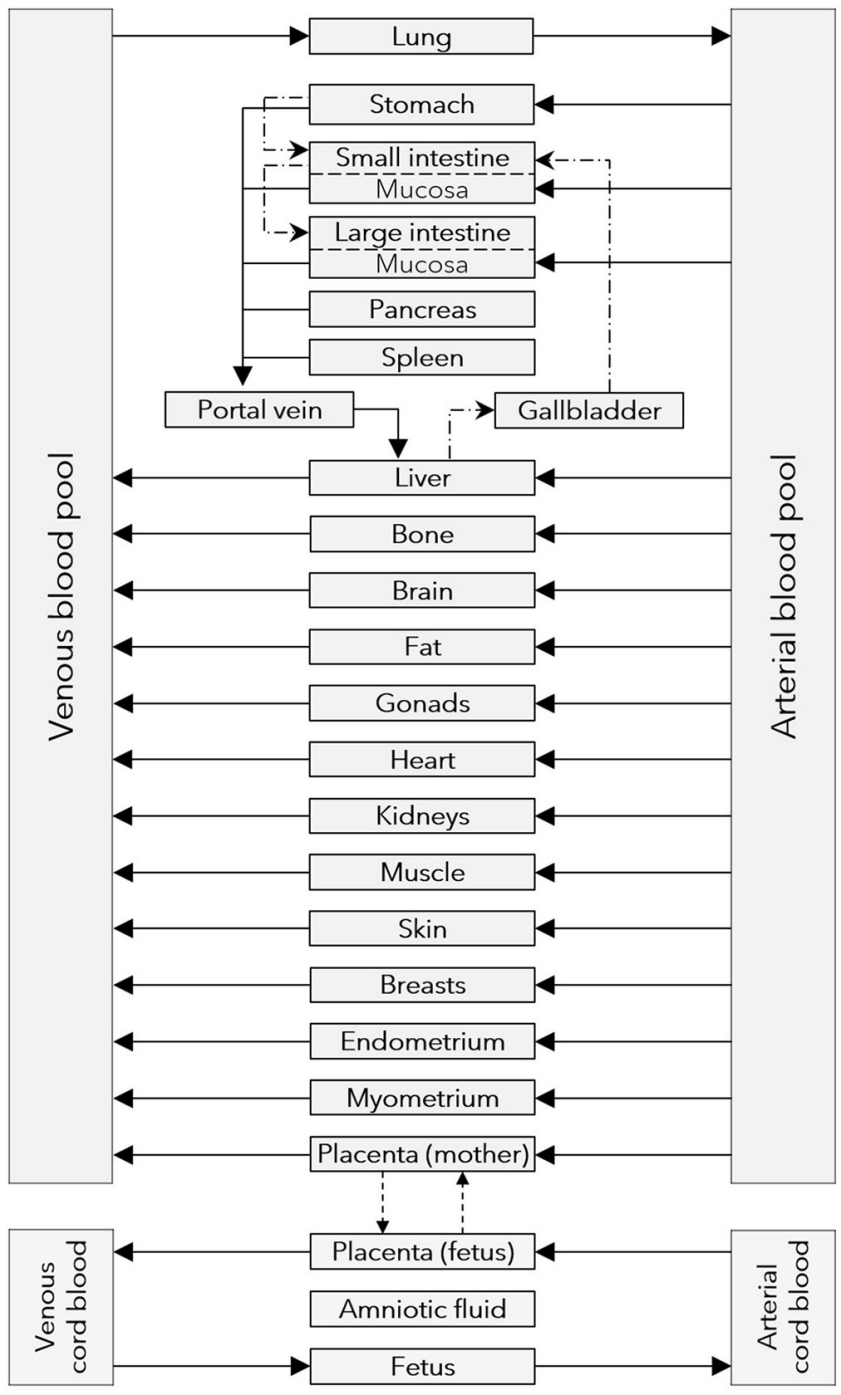
Structure of the maternal-fetal PBPK model. Gray boxes represent compartments of the physiologically based pharmacokinetic (PBPK) model; solid arrows denote drug transport via the organ blood flow; dashed arrows denote drug transport via passive diffusion; dash dotted lines denote drug transport via gastrointestinal motility or the biliary excretion route.

**Figure 2:**
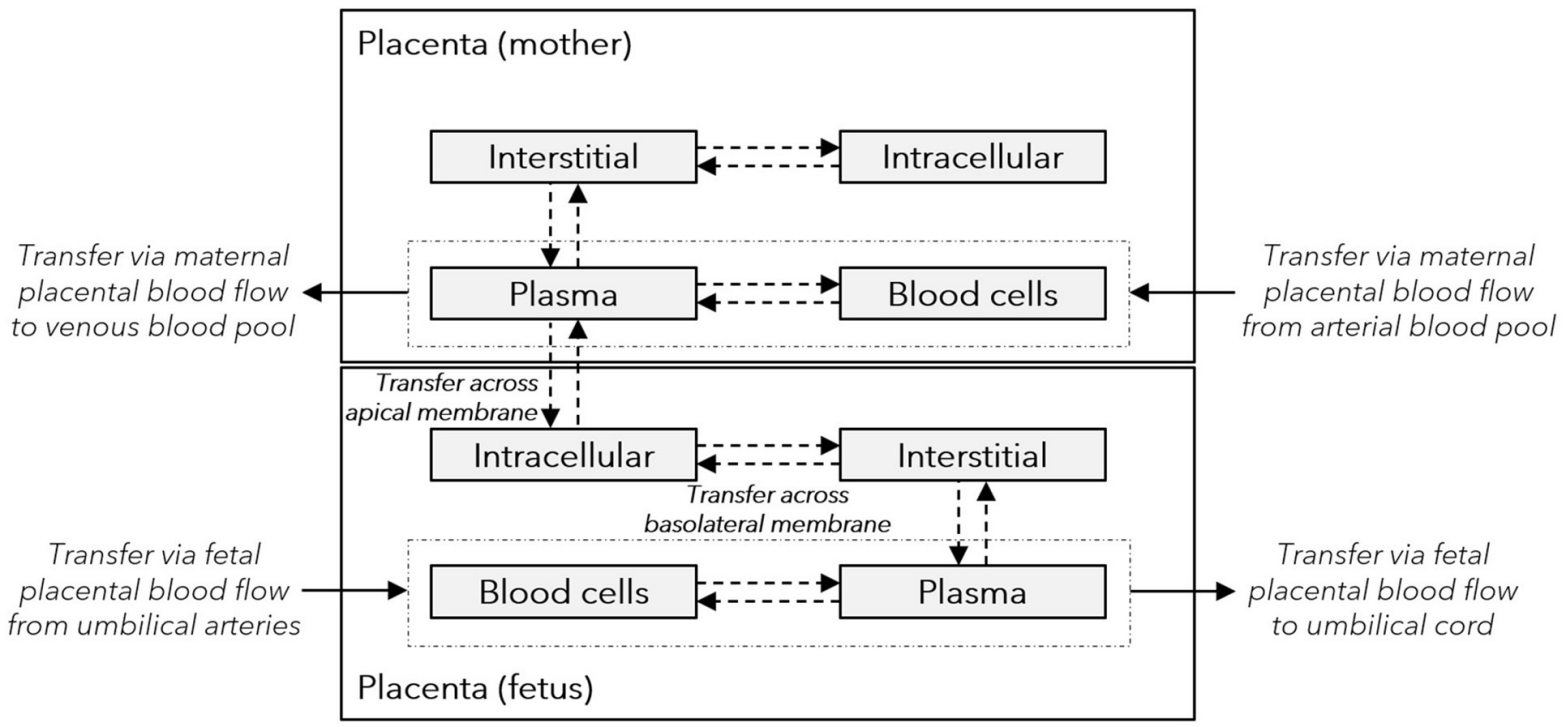
Structure of the placenta sub-model integrated in the maternal-fetal PBPK model. Gray boxes represent sub-compartments of the placenta structure implemented in the maternal-fetal physiologically based pharmacokinetic (PBPK) model; dash-dotted boxes represent the vascular space; solid arrows denote drug transport via the organ blood flow; and dashed arrows denote drug transport via passive diffusion. The maternal plasma and blood cell compartments represent the intervillous space, and the maternal interstitial and intracellular space represent the placental septae and the decidua basalis, respectively. The fetal intracellular compartment represents the (syncytio-)trophoblasts with the apical membrane facing the maternal plasma compartment and the basolateral membrane facing the fetal interstitial compartment. The fetal interstitial space represents intravillous fibrous tissue and the plasma and blood cell compartments represent the intravillous vascular system.

In the obstetric clinical pharmacology field, pharmacokinetic data in pregnant women are sparse and data sharing can be an important step to advance the development and validation of PBPK models. Here, we used data on acetaminophen pharmacokinetics in the umbilical cord from two clinical studies (17, 18) to re-evaluate and refine a recently developed maternal-fetal PBPK model (13, 19). In our previous work, acetaminophen transfer across the placenta in the PBPK model was informed based on published data from the *ex vivo* cotyledon perfusion assay (20). Therefore, an *in silico* cotyledon perfusion model was previously developed to learn the transfer kinetics in the *ex vivo* cotyledon perfusion assay before implementing them in the PBPK model. However, the previously developed *in silico* cotyledon perfusion model consisted of 4 compartments only, namely the maternal and fetal reservoir and the maternal and fetal tissue portions of the cotyledon (13). It was therefore structurally different than the placental sub-structure implemented in the PBPK model (see Figures 1, 2) (19). Consequently, the parameters of the former model are not directly transferable to the latter and hence translatability across these models may not be guaranteed.

To this end, a novel *in silico* cotyledon perfusion model was developed herein that constitutes a congruent, albeit minimized, replicate of the placental structure implemented in the maternal-fetal PBPK model. Additionally, it was intended that the novel *in silico* cotyledon perfusion model better reflected the cotyledon physiology so that potential drug accumulation in the tissue could be considered in the simulations. Several parameters of the *in silico* cotyledon perfusion model relevant to maternal-fetal drug transfer were then optimized using previously published data for acetaminophen. The optimization results were then transferred into the PBPK model and the predicted pharmacokinetics in the umbilical vein at delivery were re-evaluate using the pooled data of Nitsche et al. (17) and Mehraban et al. (18).

## Materials and Methods

### Software

PBPK models were built with PK-Sim® and MoBi® which are available as open source tools through the Open Systems Pharmacology (OSP) software, version 9.1, via GitHub (https://github.com/Open-Systems-Pharmacology) (21). The software R, version 3.6.3 (R Foundation for Statistical Computing, http://www.r-project.org) was used for graphics creation and statistical analysis. The R-package “ospsuite”, version 9.0.79 (https://github.com/Open-Systems-Pharmacology/OSPSuite-R), was used to conduct pharmacokinetic simulations in virtual populations of pregnant women. The tool WebPlotDigitizer, version 4.0 (https://automeris.io/WebPlotDigitizer/) was used to extract data from figures and conversion into numerical format.

### General Workflow

In our previous study, we successfully translated an adult, non-pregnant PBPK model for acetaminophen including its metabolites generated by uridine 5′-diphospho-glucuronosyltransferase (UGT) 1A1, sulfotransferase (SULT) 1A1 and cytochrome-P-450 (CYP) 2E1 to pregnancy (13, 19). The predicted acetaminophen pharmacokinetics in the maternal blood were previously verified in the first trimester with clinical data from Beaulac-Baillargeon and Rocheleau (22); predicted acetaminophen pharmacokinetics in the maternal and umbilical vein blood at delivery were previously evaluated with data from Allegaert et al. (23) and Nitsche et al. (17), respectively.

In this study, we developed a novel *in silico* cotyledon perfusion model that is structurally equivalent with the placenta implemented in the PBPK model. We used this *in silico* cotyledon perfusion model to learn placental transfer kinetics of acetaminophen by fitting relevant model parameters to observed data obtained in the *ex vivo* perfusion cotyledon assay (20). Once this model captured the observed *ex vivo* kinetics adequately, the values of relevant model parameters were copied to the maternal-fetal PBPK model. Note that the structure of the PBPK model was not changed in this study and is thus consistent with the structure of the PBPK model reported in our previous publication (13). Thereafter, fetal pharmacokinetics were predicted in the umbilical vein compartment of the PBPK model and predictions were evaluated with clinical data from Nitsche et al. (17) and Mehraban et al. (18).

### *In silico* Cotyledon Perfusion Model

#### Description of the Model

**Figure 3:**
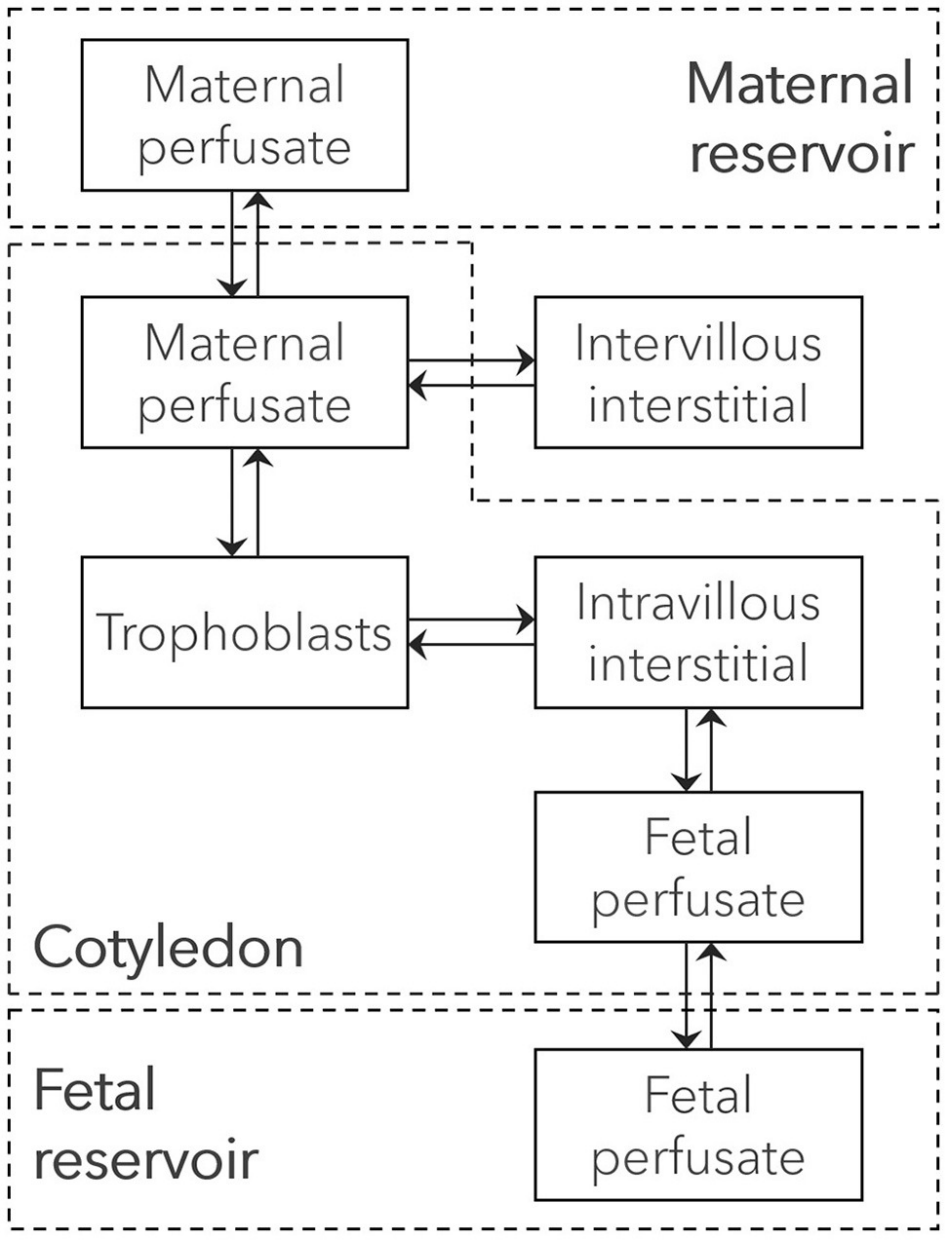
Structure of the *ex vivo* cotyledon perfusion model. Boxes represent compartments of the novel *ex vivo* cotyledon perfusion model and solid arrows denote drug transport via the perfusate flow or diffusion. The cotyledon is highlighted as dashed box.

A novel *in silico* cotyledon perfusion model structure, schematically depicted in Figure 3, was developed that closely reflects the physiological structure of the cotyledon *ex vivo*. Note that the maternal intracellular compartment representing mainly the decidua basalis is only present in the PBPK model, but not in the *in silico* cotyledon perfusion model. Since the decidua is shed off during childbirth, it is not part of the cotyledon tissue used in the *ex vivo* experiment and only present in utero. Although there will still be decidual remnants on the delivered placenta, they come off during flushing and rinsing for experimental preparation. Hence, the *in silico* cotyledon perfusion model consists of seven compartments: The maternal perfusate in the maternal reservoir and in the intervillous space of the cotyledon; the intervillous interstitial compartment representing fibrous tissue adhering to the cotyledon on the maternal-facing side; the trophoblasts; the interstitial space representing fibrous tissue in the fetal villous; and the fetal perfusate in the fetal villous and fetal reservoir. Table 1 gives an overview of the tissue components present *in vivo/ex vivo* and their corresponding compartments in the *in silico* cotyledon perfusion model (Figure 3) and the placental sub-structure of the PBPK model (Figure 2).

**Table 1:** Overview of different tissue components and their corresponding compartments in the novel *in silico* cotyledon perfusion model and the PBPK model.

**Physiological tissue component**	**Compartment name in the novel *in silico* cotyledon perfusion model**	**Compartment name in the PBPK model**
Maternal blood in the intervillous space of the cotyledon	Maternal perfusate in the cotyledon	Plasma and blood cells in the maternal part of the placenta
Placental septae	Intervillous interstitial	Interstitial space in the maternal part of the placenta
Decidua basalis	*NA* ^a^	Intracellular space in the maternal part of the placenta
(Syncytio)trophoblasts	Trophoblasts	Intracellular space in the fetal part of the placenta
Fibrous tissue in the fetal villi	Intravillous interstitial	Interstitial space in the fetal part of the placenta
Fetal blood in the blood capillaries of the fetal villi	Fetal perfusate in the cotyledon	Plasma and blood cells in the fetal part of the placenta

a *During childbirth the decidua basalis is shed off and hence not present in the ex vivo cotyledon*.

The ordinary differential equations (ODE) given in the following were used in the novel *in silico* cotyledon perfusion model. Note that in MoBi®, the ODEs are first defined for intercompartmental exchange processes in the passive transports building block; during set-up of a simulation, the ODEs are then generated for each compartment. In the following, the ODEs are first introduced for each intercompartmental exchange transport and then defined for the compartments.

The following equations were used to describe drug transfer in perfusate between the maternal reservoir and the cotyledon (Equation 1) and between the fetal reservoir and the cotyledon (Equation 2):


(1) $$\begin{array}{l} \frac{dN_{M\_perf}}{dt}=Q_{M}\times \left(C_{M\_res}-C_{M\_perf}\right) \end{array}$$

(2) $$\begin{array}{l} \frac{dN_{F\_perf}}{dt}=Q_{F}\times \left(C_{F\_res}-C_{F\_perf}\right) \end{array}$$

Here, *N*_*M*_*perf*_ and *N*_*F*_*perf*_ denote the molar drug amount in the maternal and fetal perfusate, respectively, that fills the intervillous space of the cotyledon [μmol]; *Q*_*M*_ and *Q*_*F*_ denote the flow rate of the perfusate in maternal and fetal system, respectively [L/min]; *C*_*M*_*res*_ and *C*_*F*_*res*_ the molar drug concentration in maternal and fetal perfusate in the reservoir, respectively [μmol/L]; and *C*_*M*_*perf*_ and *C*_*F*_*perf*_ the molar drug concentration in maternal and fetal perfusate in the cotyledon, respectively [μmol/L]. *Q*_*M*_ and *Q*_*F*_ as well as the volumes of the maternal and fetal reservoirs were set to the values reported by Conings et al. (20); 14 mL/min and 6 mL/min for the flow rate in the maternal and fetal system, respectively; and 280 and 284 mL for the maternal and fetal reservoir volume. Drug amount was converted to drug concentration by dividing the drug amount by the compartment's volume.

Drug transfer between maternal perfusate in the cotyledon and intervillous interstitial space and fetal perfusate in the cotyledon and intravillous interstitial space was described by Equations 3, 4, respectively:


(3) $$\begin{array}{l} \frac{dN_{M\_int}^{M\_int↔M\_perf}}{dt}=f_{u}\times P_{endo}\times SA_{M\_perf\;:\;int}\times \left(C_{M\_perf}-\frac{C_{M\_int}}{K_{M\_int\;:\;perf}}\right) \end{array}$$

(4) $$\begin{array}{l} \frac{dN_{F\_int}^{F\_int↔F\_perf}}{dt}=f_{u\_fetus}\times P_{endo}\times SA_{F\_perf\;:\;int}\times \left(C_{F\_perf}-\frac{C_{F\_int}}{K_{F\_int\;:\;perf}}\right) \end{array}$$

Here, $N_{M\_int}^{M\_int↔M\_perf}$ denotes molar drug amount in the intervillous interstitial compartment when drug exchange is only considered to occur between the intervillous interstitial space and maternal perfusate in the cotyledon [μmol]; $N_{F\_int}^{F\_int↔F\_perf}$ denotes molar drug amount in the intravillous interstitial compartment when drug exchange is only considered to occur between the intravillous interstitial space and fetal perfusate in the cotyledon [μmol]; *f*_*u*_ and *f*_*u*_*fetus*_ denote the drug's fraction unbound in maternal and fetal perfusate, respectively; *P*_*endo*_ is the drug's permeability through the endothelial membrane of blood vessels and was assumed to be equal for maternal and fetal endothelial membranes [dm/min]; *SA*_*M*_*perf* : *int*_ and *SA*_*F*_*perf* : *int*_ denote the surface area between maternal perfusate and intervillous interstitial space and fetal perfusate and intravillous interstitial space, respectively [dm^2^]; *C*_*M*_*int*_ and *C*_*F*_*int*_ the molar drug concentration in intervillous and intravillous interstitial space of the cotyledon, respectively [μmol/L]; *K*_*M*_*int* : *perf*_ is the intervillous interstitial-to-perfusate partition coefficient of the drug; and *K*_*F*_*int* : *perf*_ is the intravillous interstitial-to-perfusate partition coefficient of the drug.

The unbound fraction of acetaminophen was scaled from an adult value of 0.82 (19) as described previously (24). The bovine serum albumin concentrations present in the maternal and fetal perfusate of the *ex vivo* cotyledon perfusion assay were 40 and 30 mg/mL, respectively (20). This resulted in an unbound fraction of 0.84 and 0.88 in maternal and fetal perfusate, respectively. Permeability across the endothelial membrane in the intervillous and intravillous space (*P*_*endo*_) was assumed to be not the rate-limiting step for tissue distribution and was hence set to a value of 100 cm/min as has been done for other organ compartments (except the brain) in whole-body PBPK models (24). Note that for drugs that are substrates to efflux transporters expressed in the endothelial membrane, this rate may have to be reduced. *SA*_*M*_*perf* : *int*_ and *SA*_*F*_*perf* : *int*_ were estimated by scaling the local surface area from the cotyledon volume assuming that organ structure is geometrically similar among species (24). The volumes of the intervillous and intravillous cotyledon fraction were assumed to be 23 and 35 mL, respectively (25). *K*_*M*_*int* : *perf*_ was calculated from the biochemical tissue composition of the cotyledon and the drug's physicochemical properties using the equation published by Schmitt (26). Values for the biochemical composition of the placenta have been reported previously (27). Finally, *K*_*F*_*int* : *perf*_ was calculated accordingly, except that the fraction unbound in the original equation by Schmitt (26) was replaced by the fetal fraction unbound (Equation 5):


(5) $$\begin{array}{l} K_{F\_int\;:\;perf}=\left(f_{water}^{int}+\frac{f_{protein}^{int}}{f_{protein}^{perf}}\times \left(\frac{1}{f_{u\_fetus}}-f_{water}^{perf}\right)\right)\times f_{u\_fetus} \end{array}$$

In this equation, $f_{water}^{int}$ and $f_{water}^{perf}$ denote the fractional water content in intravillous interstitial space and perfusate, respectively; and $f_{protein}^{int}$ and $f_{protein}^{perf}$ the fractional protein content in intravillous interstitial space and perfusate, respectively. $f_{water}^{int}$ was assumed to be the same than for the intervillous interstitial space [0.935 (28)] and $f_{water}^{perf}$ was assumed to be similar to the fractional volume content reported for plasma [0.926 (28)]. The value for the ratio $\frac{f_{protein}^{int}}{f_{protein}^{perf}}$ in the *ex vivo* cotyledon was assumed to be the same as in adult tissue [0.37 (26)].

Intravillous drug transfer between interstitial and intracellular space (i.e., the trophoblasts) was described by Equation 6:


(6) $$\begin{array}{l} \frac{dN_{F\_cell}^{F\_cell↔F\_int}}{dt}\;=\;P\times SA_{F\_int\;:\;cell}\times \\ \;(K_{F\_water\;:\;int}\times C_{F\_int}-\;K_{F\_water\;:\;cell}\times C_{F\_cell}) \end{array}$$

Here, $N_{F\_cell}^{F\_cell↔F\_int}$ denotes molar drug amount in the intracellular space when drug exchange is only considered to occur between the intracellular and intravillous interstitial compartment [μmol]; *P* is the drug's membrane permeability [dm/min] which was calculated from the drug's effective molecular weight and lipophilicity as described elsewhere (24); *SA*_*F*_*int* : *cell*_ is the surface area between the intravillous interstitial space and the intracellular space (i.e. trophoblasts) [dm^2^]; *K*_*F*_*water* : *int*_ the partition coefficient between water and intravillous interstitial space; *K*_*F*_*water* : *cell*_ the partition coefficient between water and intracellular space of the trophoblasts; and *C*_*F*_*cell*_ the molar drug concentration in the trophoblasts [μmol/L]. The local surface area in this equation was calculated as already described above. *K*_*F*_*water* : *int*_ and *K*_*F*_*water* : *cell*_ were expressed as follows:


(7) $$\begin{array}{l} K_{F\_water\;:\;int}=\frac{f_{u\_fetus}}{K_{F\_int\;:\;perf}} \end{array}$$

(8) $$\begin{array}{l} K_{F\_water\;:\;cell}=\frac{f_{u}\_fetus}{K_{F\_cell\;:\;perf}} \end{array}$$

where *K*_*F*_*int* : *perf*_ is calculated according to the Equation 5 and *K*_*F*_*cell* : *perf*_ according to the cell-to-plasma partition coefficient equation. In the developed *in silico* cotyledon perfusion model, several equations were implemented to calculate these partition coefficients from the biochemical tissue composition of the placenta (27) and the drug's physicochemical properties using, namely the PK-Sim Standard equation (29) and the equations proposed by Schmitt et al. (26), Rodgers et al. (30, 31), and Poulin et al. (32, 33). For acetaminophen, the partition coefficient equation by Rodgers et al. (30, 31) was used. Note that in the presented model, *K*_*F*_*cell* : *perf*_ was included as global parameter that used per default the maternal fraction unbound; to correct for the fetal fraction unbound, *K*_*F*_*cell* : *perf*_ was multiplied by the ratio of fetal-to-maternal fraction unbound. Hence, inserting Equations 5, 7, 8 into Equation 6 as well as correcting for the fetal fraction unbound yields Equation 9 which was implemented in the model:


(9) $$\begin{array}{l} \frac{dN_{F\_cell}^{F\_cell↔F_{i}nt}}{dt}=P\times SA_{F\_int\;:\;cell}\\ \times \left(C_{F\_int}\times {\left(f_{water}^{int}+\frac{f_{protein}^{int}}{f_{protein}^{perf}}\times \left(\frac{1}{f_{u\_fetus}}-f_{water}^{perf}\right)\right)}^{-1}\right)\\ \left(-C_{F\_cell}\times \frac{f_{u}\_fetus}{K_{F\_cell\;:\;perf}\times \frac{f_{u\_fetus}}{f_{u}}}\right) \end{array}$$

Or, alternatively and in a shorter form (Equation 10):


(10) $$\begin{array}{l} \frac{dN}{dt}\;=\;P\times SA_{F\_int\;:\;cell}\\ \;\times \left(C_{F\_int}\times \frac{f_{u\_fetus}}{K_{F\_int\;:\;perf}}-C_{F\_cell}\times \frac{f_{u}}{K_{F\_cell\;:\;perf}}\right) \end{array}$$

Finally, maternal-fetal drug transfer across the apical side of the trophoblast was modeled between maternal perfusate in the intervillous space and the trophoblasts using Equation 11:


(11) $$\begin{array}{l} \frac{dN_{F\_cell}^{F\_cell↔M\_perf}}{dt}\;=\;P\times SA_{villi}\times f_{u}\\ \;\times \left(f_{in}\times C_{M\_perf}-f_{out}\times \frac{C_{F\_cell}}{K_{FM\_cell\;:\;perf}}\right) \end{array}$$

In this equation, $N_{F\_cell}^{F\_cell↔M\_perf}$ denotes molar drug amount in the intracellular space when drug exchange is only considered to occur between the intracellular space and maternal perfusate in the cotyledon [μmol]; *SA*_*villi*_ is the surface area of the fetal villi at the interface of maternal perfusate in the cotyledon and trophoblasts [dm^2^]; *f*_*in*_ and *f*_*out*_ are factors modifying the influx and efflux permeability of the drug (i.e. in maternal→fetal and fetal→maternal direction), respectively; and *K*_*FM*_*cell* : *perf*_ is the drug's partition coefficient between fetal intracellular space (trophoblasts) and maternal perfusate in the cotyledon.

Per default, *f*_*in*_ and *f*_*out*_ in Equation 11 are set to 1 (i.e. equal permeability in both directions). *SA*_*villi*_ was estimated by diving the absolute surface area of all fetal villi in the term placenta, ~1178 dm^2^ (27), by the average number of cotyledons in the placenta which varies around 35 at term (34). The drug's permeability across the trophoblasts' membrane was calculated from the drug's effective molecular weight and lipophilicity as described elsewhere (24) resulting in a value of 4.29 · 10^−2^ cm/min for acetaminophen. *K*_*FM*_*cell* : *perf*_ was estimated as described above, i.e. according to the method described by Rodgers et al. (30, 31). Of note, the value of *K*_*FM*_*cell* : *perf*_ in Equation 11 is similar to that of *K*_*F*_*cell* : *perf*_ in Equation 10 because both partition coefficients refer to the same intracellular compartment (trophoblast). *K*_*FM*_*cell* : *perf*_ is located at the apical membrane and *K*_*F*_*cell* : *perf*_ at the basolateral membrane of the trophoblasts.

Hence, combining the ODEs above for the specific intercompartmental exchange processes gives the full ODE system of the novel *in silico* cotyledon perfusion model:


(12) $$\begin{array}{l} dt\left[\begin{array}{c} N_{M\_res}\\ N_{M\_perf}\\ N_{M\_int}\\ N_{F\_cell}\\ N_{F\_int}\\ N_{F\_perf}\\ N_{F\_res} \end{array}\right]=E\left[\begin{array}{c} C_{M\_res}\\ C_{M\_perf}\\ C_{M\_int}\\ C_{F\_cell}\\ C_{F\_int}\\ C_{F\_perf}\\ C_{F\_res} \end{array}\right] \end{array}$$

Here, *N* and *C* denote molar drug amount [μmol] and molar drug concentration [μmol/L] in the compartment specified by the subscript and *E* describes the intercompartmental drug exchange processes that have been specified in Equations 1–11. More specifically, *E* can be written as the following 7 × 7 matrix:


(13) $$\begin{array}{l} E=\left[\begin{array}{ccccccc} -Q_{M} & Q_{M} & 0 & 0 & 0 & 0 & 0\\ Q_{M} & -Q_{M}-f_{u}\times \left(P_{endo}\times SA_{M\_perf\;:\;int}+P\times SA_{villi}\times f_{in}\right) & P_{endo}\times SA_{M\_perf\;:\;int}\times f_{u} & P\times SA_{villi}\times f_{out}\times \frac{f_{u}}{K_{F\_cell\;:\;perf}} & 0 & 0 & 0\\ 0 & P_{endo}\times SA_{M\_perf\;:\;int}\times f_{u} & P_{endo}\times SA_{M\_perf\;:\;int}\times \frac{f_{u}}{K_{M\_int\;:\;perf}} & 0 & 0 & 0 & 0\\ 0 & P\times SA_{villi}\times f_{in}\times f_{u} & 0 & -P\times f_{u}\times \left(f_{out}\times \frac{SA_{villi}}{K_{FM\_cell\;:\;perf}}+\frac{SA_{F\_int\;:\;cell}}{K_{F\_cell\;:\;perf}}\right) & P\times SA_{F\_int\;:\;cell}\times \frac{f_{u\_fetus}}{K_{F\_int\;:\;perf}} & 0 & 0\\ 0 & 0 & 0 & P\times SA_{F\_int\;:\;cell}\times \frac{f_{u}}{K_{F\_cell\;:\;perf}} & -\frac{f_{u\_fetus}}{K_{F\_int\;:\;perf}}\times \left(P\times SA_{F\_int\;:\;cell}+P_{endo}\times SA_{F\_perf\;:\;int}\right) & P_{endo}\times SA_{F\_perf\;:\;int}\times f_{u\_fetus} & 0\\ 0 & 0 & 0 & 0 & P_{endo}\times SA_{F\_perf\;:\;int}\times \frac{f_{u\_fetus}}{K_{F\_int\;:\;perf}} & -Q_{F}-P_{endo}\times SA_{F\_perf\;:\;int}\times f_{u\_ftus} & 0\\ 0 & 0 & 0 & 0 & 0 & Q_{F} & -Q_{F} \end{array}\right] \end{array}$$

#### Model Optimization

After the model has been implemented as described above, it was used to simulate acetaminophen concentrations in the maternal and fetal reservoir. Model parameters relevant for placental drug transfer, namely *f*_*in*_, *f*_*out*_ and the placental partition coefficients (*K*_*FM*_*cell* : *perf*_ on the apical side and *K*_*F*_*cell* : *perf*_ on the basolateral side of the trophoblast), were optimized to better capture the observed data reported by Conings et al. (20). More specifically, the following optimization scenarios were tested:

- Optimizing symmetrical drug transfer: *f*_*in*_ and *f*_*out*_ were both fitted together so that the permeability in maternal→fetal and fetal→maternal direction was modified by the same factor.
- Optimizing asymmetrical drug transfer: *f*_*in*_ and *f*_*out*_were fitted separately from each other, resulting in different permeability values in maternal→fetal and fetal→maternal direction.
- Optimizing symmetrical drug transfer and the placental partition coefficients: In addition to fitting *f*_*in*_ and *f*_*out*_ together, *K*_*FM*_*cell* : *perf*_ and *K*_*F*_*cell* : *perf*_ were also fitted together (i.e. both partition coefficients had the same fitted value). Hence, the permeability in maternal→fetal and fetal→maternal direction was modified by the same factor as were the partition coefficients. Fitting *K*_*FM*_*cell* : *perf*_ and *K*_*F*_*cell* : *perf*_ facilitated changes in intracellular drug concentrations (e.g., causing drug accumulation in the trophoblasts).
- Optimizing asymmetrical drug transfer and the placental partition coefficients: *f*_*in*_ and *f*_*out*_ were fitted separately from each other, while *K*_*FM*_*cell* : *perf*_ and *K*_*F*_*cell* : *perf*_ were fitted together (i.e. both partition coefficients had the same fitted value). This resulted in different permeability values in maternal→fetal and fetal→maternal direction and in modified additionally intracellular drug concentrations.

Parameter optimizations were conducted using the built-in module in MoBi® and the Monte-Carlo algorithm. All observed data [in total 18 data sets comprising 455 data values (20)] were included in the parameter optimization.

### Fetal-Maternal PBPK Model for Acetaminophen

#### Description of the Model

After training the novel *in silico* cotyledon perfusion model to learn placental transfer kinetics of acetaminophen from the *ex vivo* cotyledon perfusion data, the fitted parameter values were copied to the PBPK model to predict acetaminophen pharmacokinetics in the umbilical vein at delivery. The structure of this whole-body model is schematically shown in Figure 1 and the sub-structure of the placenta implemented in that model is depicted in Figure 2. In this placental sub-structure, the maternal plasma and blood cell compartments represent together the intervillous space, the maternal interstitial represents the placental septae and the maternal intracellular space represents the decidua basalis which is distorted during labor-induced contraction of the myometrium and eventually destroyed by the hemorrhages during delivery. The fetal intracellular compartment represents the (syncytio-)trophoblasts with the apical membrane facing the maternal plasma compartment and the basolateral membrane facing the fetal interstitial compartment. The fetal interstitial space represents intravillous fibrous tissue and the plasma and blood cell compartments represent the intravillous vascular system.

The PBPK model was corrected for the drug's unbound fraction in fetal compartments. Unbound maternal and fetal fraction of acetaminophen were calculated from the albumin plasma concentrations using a previously reported function (24). For the mother, albumin plasma concentrations were calculated for each patient's gestational age (27), while for the fetus, a concentration of 32 g/L was used as has been reported for the gestational age range from 35 to 38 weeks (35). Placental transfer parameters in the PBPK model were replaced with the fitted values from the *in silico* cotyledon perfusion model (see section Model Optimization). Of note, while the permeability across the placenta in the PBPK model was the same as in the *in silico* cotyledon perfusion model (4.29 · 10^−2^ cm/min), the transfer rate in the PBPK model was scaled with the villi surface area resulting thus in a larger transfer clearance for the total placenta compared to the cotyledon. All other model parameters were kept the same as published previously (13, 19).

#### Model Simulations

Pharmacokinetics were predicted in the venous plasma of the umbilical cord. For each patient, a virtual population of 100 pregnant women with the patient's body weight and height as well as gestational week was created using the population creation algorithm of the R-package ‘ospsuite'. Unfortunately, the study by Nitsche et al. (17) did not report the individual body weight and height of each patient and, thus, a virtual population with standard body weight at term delivery (24) was used for the simulations. The population simulation results for all patients were pooled for calculation of the overall median and percentiles and for further analysis.

#### Patients and Data

Nitsche et al. (17) studied maternal and fetal pharmacokinetics in 34 women without medical or obstetrical complications following a single oral dose of 1,000 mg acetaminophen upon admission for scheduled cesarean delivery. Patient characteristics are listed in Table 2. Pharmacokinetic data were extracted from the concentration-time profile figure published by Nitsche et al. (17). From the 34 women, only 28 data values could be extracted.

**Table 2:** Patient characteristics.

	**Study by Nitsche et al. (17)**	**Study by Mehraban et al. (18)**
No. of patients	34	43
Maternal age [years]	32 [25–39]	30 [20–35]
Maternal weight [kg]	82 [62–100]	76 [46–136]
Maternal height [cm]	*not reported*	178 [149–209]
Gestational age at delivery [weeks]	39 [38–40]	39 [37–41]

*Data are expressed as median [range]*.

Additional concentration data in the umbilical cord were obtained from the study by Mehraban et al. (18). From the 121 patients with intrapartum fever of whom blood samples were collected in this study, we included the 45 patients who received a single oral dose of 1,000 mg acetaminophen. Two additional patients were excluded from our analysis because of unusually high acetaminophen concentrations in the umbilical cord which we attributed to documentation errors (the umbilical vein concentrations of these patients were 14.5 and 3.1 mg/L at 18 and 55 h after dose administration). Characteristics of the patients included in our analysis are listed in Table 2.

Few measured concentrations in the study by Mehraban et al. (18) fell below the lower limit of quantification (LLOQ). These data were included as LLOQ/2 in this analysis. It has to be noted that in the study by Mehraban et al. (18), maternal concentrations were not measured.

#### PBPK Model Evaluation

Predicted concentrations in the umbilical vein were visually compared to clinical data obtained from clinical studies. Additionally, goodness-of-fit plots depicting predicted vs. observed concentrations and residuals vs. time plots were created and the mean prediction error (MPE) and mean absolute prediction error (MAPE) were calculated.

### Ethics

Ethics and study registration related aspects are clearly mentioned in the original publications (17, 18) that served as source of this refinement effort, and no additional registration or procedures were needed.

## Results

### *In silico* Cotyledon Perfusion Model

Of the four tested optimization scenarios, optimizing asymmetrical drug transfer and the placental partition coefficients yielded the lowest simulation error. The fitted value ± 95% confidence interval for the placental partition coefficients, *K*_*FM*_*cell* : *perf*_ and *K*_*F*_*cell* : *perf*_, was 4.31 ± 0.57 [vs. 0.76 when being estimating according to the method described by Rodgers et al. (30, 31)] resulting in a substantial amount of acetaminophen accumulating in the trophoblasts of the cotyledon.

**Figure 4:**
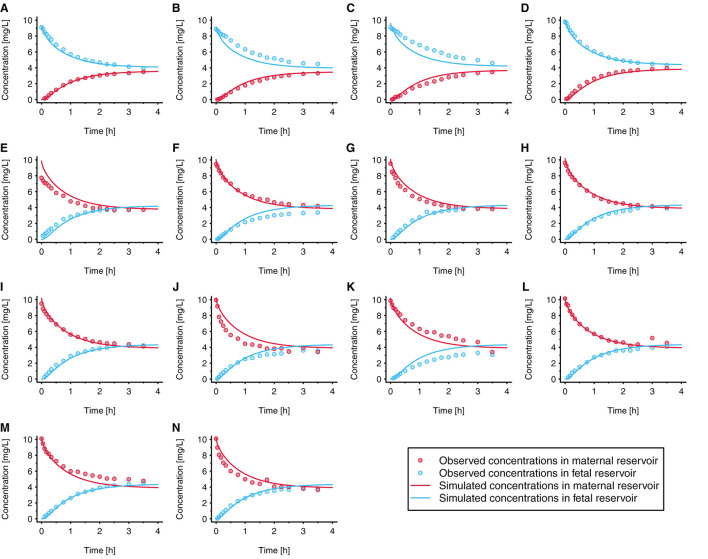
Observed and simulated concentration time profiles in the novel *ex vivo* cotyledon perfusion model. In each experiment, acetaminophen was either administered to the fetal reservoir **(A–D)** or maternal reservoir **(E–N)** at time = 0h. The initial concentration was 10 mg/L in all experiments (to reflect clinically relevant concentrations); each panel refers to an individual experiment. All experiments were conducted under similar conditions. Observed data were taken from Conings et al. (20).

The fitted values ± 95% confidence intervals for *f*_*in*_ and *f*_*out*_ were 0.060 ± 0.0058 and 0.051 ± 0.0061, respectively. This resulted in permeability values in maternal→fetal and fetal→maternal direction of 2.56 × 10^−3^ and 2.18 × 10^−3^ cm/min, respectively, vs. 4.29 × 10^−2^ cm/min when the permeability was estimated from the physicochemical properties of acetaminophen. Figure 4 presents the observed and simulated concentration-time profiles of acetaminophen in maternal and fetal perfusate in the reservoirs of the *ex vivo* cotyledon perfusion assay.

**Figure 5:**
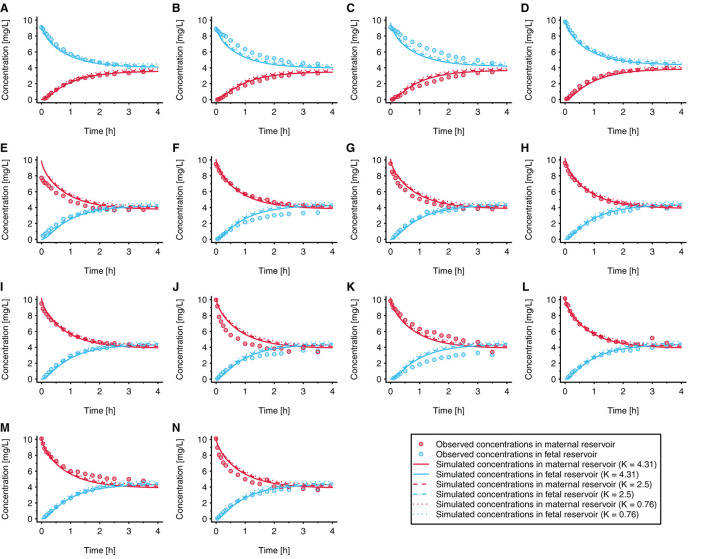
Local sensitivity analysis for the placental partition coefficients (*K*_*FM*_*cell* : *perf*_ and *K*_*F*_*cell* : *perf*_). In each experiment, acetaminophen was either administered to the fetal reservoir **(A–D)** or maternal reservoir **(E–N)** at time = 0 h. The initial concentration was 10 mg/L in all experiments (to reflect clinically relevant concentrations); each panel refers to an individual experiment. All experiments were conducted under similar conditions. Observed data were taken from Conings et al. (20).

The results of a local sensitivity analysis are presented in Figure 5. In this figure, simulation results are shown when *K*_*FM*_*cell* : *perf*_ and *K*_*F*_*cell* : *perf*_ were set to 0.76 [i.e., the value estimated according to the method described by Rodgers et al. (30, 31)], 2.5, and 4.31 (i.e., the fitted value). All other model parameter values were kept unchanged in this sensitivity analysis. Pooled over all individual experiments, the MPE was 375%, 131, and −62.6% when using placental partition coefficient values of 0.76, 2.5, and 4.31, respectively.

### PBPK Model Evaluation

**Figure 6:**
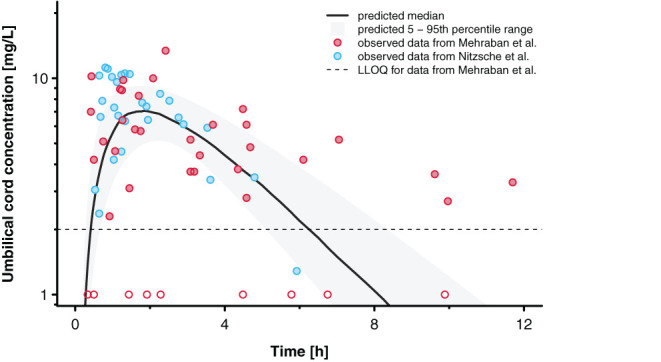
Concentration time profiles for acetaminophen in the umbilical cord. Blue and red circles indicate observed clinical data reported by Nitsche et al. (17) and Mehraban et al. (18). Data from Mehraban et al. (18) below the lower limit of quantification (LLOQ), shown as dashed line, are included as LLOQ/2 and empty circles in the figure. The black line indicates the predicted median and the shaded area the predicted 5–95th percentile range.

Figure 6 presents the concentration-time profile predicted in the umbilical vein together with the clinical data reported by Nitsche et al. (17) and Mehraban et al. (18). The observed inter-individual variability, especially in the data reported by Mehraban et al. (18), was considerably larger than the predicted variability. For the pooled data sets, MPE and MAPE were 24.7 and 68.7%, respectively. For the data reported by Nitsche et al. (17), MPE and MAPE were −10.4 and 29.9%, respectively; and for the data reported by Mehraban et al. (18), MPE and MAPE were 49.3 and 96.0%, respectively.

**Figure 7:**
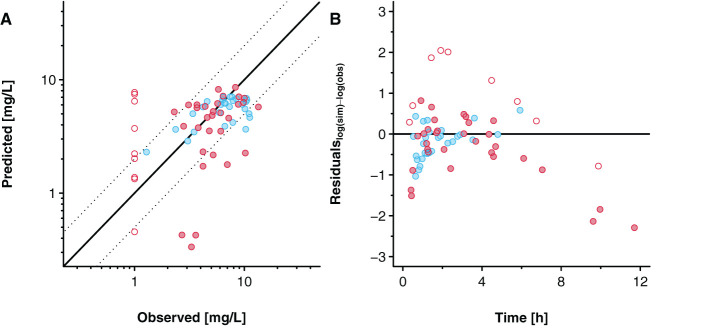
Goodness-of-fit plot **(A)** and residuals versus time plot **(B)** for the predicted acetaminophen concentrations in the umbilical vein. Blue and red circles indicate clinical data reported by Nitsche et al. (17) and Mehraban et al. (18). Data from Mehraban et al. (18) below the lower limit of quantification (LLOQ) are included as LLOQ/2 and empty circles in the figure. In **(A)**, the solid line indicates the line of identity and the dashed lines limit the 2-fold error range. In **(B)**, the solid line indicates the level where the residuals are zero.

Figure 7 presents the goodness-of-fit plot and the residuals plotted against time. For the pooled data sets, 50 (81%) out of 62 concentration values were predicted within a 2-fold error range (excluding values below LLOQ). For the data reported by Nitsche et al. (17), 25 (89%) out of 28 concentrations were predicted within the 2-fold error range, whereas for the data reported by Mehraban et al. (18), 25 (74%) out of 34 concentrations that were above LLOQ were predicted within that range.

## Discussion

The *ex vivo* cotyledon perfusion assay is often used to quantify drug transfer across the placenta and the results obtained from this assay can be leveraged in a PBPK modeling framework (36). While the previously developed *in silico* cotyledon perfusion model for acetaminophen directly links maternal with fetal perfusate (13), the PBPK model separates maternal from fetal blood plasma by interposing the fetal intracellular compartment (representing the trophoblasts) and the fetal interstitial compartment (representing stroma tissue in the fetal villi) (19). Owing to these structural differences, parameters in the *in silico* cotyledon perfusion model did not translate directly to parameters in the PBPK model. For example, the partition coefficient between maternal and fetal perfusate in the former model did not have an equivalent parameter in the PBPK model. Hence, this study aimed at developing an *in silico* cotyledon perfusion model with a more physiologic representation of the cotyledon and a compartmentalization of different tissue portions. Subsequently, this model was optimized to simulate published data for acetaminophen (20). Several parameters relevant to maternal-fetal drug transfer were optimized in this model and the best optimization results were then transferred into a previously developed whole-body PBPK model for acetaminophen (13, 19) to predict pharmacokinetics in the umbilical vein at delivery. The predictive performance of the PBPK model was then assessed with clinical data reported by Nitsche et al. (17) and Mehraban et al. (18).

The developed *in silico* cotyledon perfusion model consisted of seven compartments (Figure 3). It could be argued that the fetal compartment is missing the fetal endothelial cells since they represent an additional barrier to diffusion into the fetal plasma and blood cells which may become relevant, especially when it constitutes the rate-limiting step for drug distribution into the fetal reservoir. In this case, this barrier could be technically simulated by reducing the permeability across the endothelial membrane in the intravillous space (*P*_*endo*_). This also applies to drugs that are substrates to efflux transporters expressed in the endothelial membrane. While the transfer rate across the endothelial cells could be technically simulated, drug accumulation in the endothelial cells cannot be described by this model. For this case, a structural refinement of the model is necessary.

Data measured in the *ex vivo* cotyledon perfusion assay for acetaminophen (20) were used to optimize the model so that the observed data could be adequately reproduced (Figure 4). Obviously, the volume of the cotyledon [23 and 35 mL for the intervillous and intravillous cotyledon fraction, respectively (25)] appeared not always correctly parameterized since some simulations overestimated the observed concentrations at time point zero (see e.g., Figure 4E), although experimental sampling or measurement errors could also have given rise to these deviations.

It was observed that when increasing the placental partition coefficients, steady state acetaminophen concentrations in the maternal and fetal reservoir were slightly better simulated as indicated by the lower MPE, although the overall effect of higher placental partition coefficients on the concentrations in the reservoirs was rather small (Figure 5). The results of the sensitivity analysis (Figure 5) indicated that the measured acetaminophen concentrations in the maternal and fetal reservoirs were somewhat sensitive when accumulation in the trophoblasts was increased. Hence, the information content in these data was rather limited and results should be interpreted considering this uncertainty. Even though some uncertainty with respect to acetaminophen accumulation in the trophoblasts remains, the model simulations, especially the simulated steady state concentrations in the reservoirs, improved when acetaminophen was, at least to some extent, ‘removed' from the reservoirs by either shifting it into other compartments (e.g., the trophoblasts) or completely removing it from the system. In the *ex vivo* experiment, this could have been caused by either accumulation in the cotyledon, by binding to the experimental equipment (e.g., the inner wall of the tubes) or by the loss of acetaminophen due to e.g., the sampling procedure or metabolism in the cotyledon. Here, rather than modeling acetaminophen metabolism, the partition coefficients *K*_*FM*_*cell* : *perf*_ and *K*_*F*_*cell* : *perf*_ were fitted allowing an accumulation in the trophoblasts. The fitted value (4.31) indicated that simulated acetaminophen concentrations at steady state are more than 4-fold greater in the trophoblasts vs. the maternal perfusate. Although various clinical studies have shown that several drugs accumulate in placental tissue, including ciprofloxacin (37), sildenafil (38), and tacrolimus (39), it is unclear whether acetaminophen also accumulates in placental tissue. If so, acetaminophen might affect prostaglandin synthesis in the placenta, and subsequent fetal exposure with potentially deleterious effects on fetal development, future studies may further investigate this point.

However, metabolism could, at least partly, also be an explanation why the amount of acetaminophen in the maternal and fetal perfusate at steady state was lower than initially expected. Acetaminophen is predominantly metabolized by members of the UGT1A subgroup (mainly UGT1A1), members of the SULT1A subgroup (mainly SULT1A1), whereas a very minor extent is metabolized by CYP1A2, 2E1 and 3A4 to the toxic metabolite *N*-acetyl-*p*-benzoquinone imine (NAPQI). Although expression studies show somewhat conflicting results, it appears that most of these enzymes, including UGT1A, CYP1A2, and CYP2E1, can only be detected in placentae collected in the first trimester, but not in term placentae, whereas CYP3A4 is expressed, but apparently not functionally active in the term placenta (40, 41). SULT1A was reported to be expressed and active in the term placenta (42). However, Conings et al. (20) observed that during *ex vivo* cotyledon perfusion experiments with acetaminophen, the phase II metabolites acetaminophen glucuronide and sulfate could not be detected, whereas in perfusion experiments with acetaminophen glucuronide and sulfate, back-conversion to acetaminophen (deglucuronidation and desulfation) seemed to occur. Hence, placental metabolism of acetaminophen—and the potential conversion of phase II metabolites to the parent compound—is currently poorly understood. This aspect should be further investigated in future studies to disentangle acetaminophen accumulation and metabolism in the placenta as well as formation from its phase II metabolites. Accounting (even partially) for metabolism could improve the value of the model. This issue could potentially be addressed more specifically when selecting another drug with a better characterization of its placental metabolism profile.

The presented model enables to explore the concentration-time and concentration-effect profiles in placental tissue. To illustrate the relevance of this construct, with acetaminophen as example, we should be aware that the placenta is also a highly active secreting endocrine organ. This includes prostaglandins secreted to the fetal circulation to ensure high prostaglandin exposure throughout fetal life. In the event of transient placental acetaminophen accumulation, it is likely that this will affect prostaglandin synthesis, and subsequent fetal exposure. This may provide additional insight in the side effects associated with maternal acetaminophen (neurodevelopmental, fetal patent ductus constriction, atopy, fertility) intake during pregnancy, in addition or besides the subsequent fetal acetaminophen exposure (43–46). Finally, the concept of accumulation in placental tissue as observed for different compounds, and integrated in the current PBPK model should stimulate researchers to also consider conducting *ex vivo* cotyledon perfusion studies in both naïve as well as in placentas already exposed to a given compound before delivery.

The maternal-fetal PBPK model was used in a second step to predict acetaminophen concentrations in the umbilical cord at delivery. It was assumed that, apart from the pregnancy-induced physiological changes, neither labor nor the patient's condition (e.g., intrapartum maternal fever) would influence acetaminophen pharmacokinetics. The only adjustment related to labor and/or drugs administered in the peripartum period, such as opioids, was the 3-fold increase in gastric emptying time in the model as discussed previously (13). While all patients in the dataset by Mehraban et al. (18) received epidurals, pharmacokinetic data for the mother were not measured. The maternal pharmacokinetic data reported by Nitsche et al. (17) suggest that a 3-fold increase in gastric emptying time in the PBPK model is adequate to capture the data (13). Similar findings were also reported by Mendes et al. (9), although the authors changed the absorption rate and not gastric emptying time in their PBPK model.

The prediction results indicated that acetaminophen pharmacokinetics in the umbilical vein were overall satisfactory (Figure 6). However, inter-individual variability was substantially underestimated. This findings is not surprising because the physiological variability implemented in the PBPK model was derived from healthy pregnant women who were not in labor (27). Obviously, relatively little data is available that quantifies inter-individual variability in relevant physiological parameters (gastric emptying, organ blood flows, glomerular filtration rate etc.) during labor and hence the integration of physiological variability in PBPK models can at best be driven by plausible assumptions that are subsequently evaluated with clinical data. The predicted median concentration-time profile captured the observed data reasonably, though, as indicated by the relatively small MPE of 25.9%. Future studies should hence focus on reasonably capturing the large variability. This might also help assessing whether e.g., the two patients excluded from this analysis should really be treated as outliers (or documentation errors) or whether the unusually high concentrations from these patients could be attributed to e.g., variations in placental permeability as a consequence of labor, concomitant drug intake and/or diseases.

Unfortunately, observed maternal concentration data were not available for all patients. Such data could have helped better identifying the reasons why for some patients the fetal concentrations were rather poorly predicted. For example, a relatively large proportion of the fetal concentrations reported by Mehraban et al. (18) fell below the LLOQ. Without corresponding maternal data, is it difficult to determine whether acetaminophen poorly crossed the placenta barrier in these patients or whether maternal pharmacokinetics (e.g., delayed absorption or very fast metabolism) were responsible for the low fetal concentrations. A disease-related effect on placental drug transfer might, at least to some extent, explain the low concentrations in the umbilical cord. From the 43 patients included in this analysis, three were hypertensive and four additional patients were diabetic. Both conditions appear to be associated with impaired uteroplacental blood flow (47, 48). Additionally, it has been found that placentae from pregnancies complicated by gestational diabetes, especially if poorly controlled, show a decrease in the fluidity and a thickening of the syncytiotrophoblasts' membrane (49–51). These alterations could, at least partly, reduce placental drug transfer. In fact, from the seven hypertensive or diabetic patients included in this analysis, five umbilical cord samples yielded concentrations below the LLOQ, whereas the concentrations from the other hypertensive or diabetic patients did not appear to differ from the remaining data. Interestingly, although one of the two patients excluded from this analysis was also suffering from gestational hypertension, the umbilical cord concentration obtained from this patient was unusually high (3.1 mg/L at 55 h) which seems inconsistent with a reduced placental drug transfer. Still, these considerations illustrate that the influence of diseases on the physiology in pregnant women should ideally be integrated in PBPK models, if applied to a clinical setting.

Several limitations pertain to the presented findings and models. In the *in silico* cotyledon perfusion model, many parameters could not be adequately identified, and hence biologically plausible assumptions had to be made. For example, the biochemical tissue composition of the placenta and cotyledon—a relevant input parameter for estimating the partition coefficients—was assumed to be similar. Currently, information is lacking in the scientific literature that would allow are more detailed discrimination of the biochemical composition of intervillous and intravillous structures in the placenta. Also, enzyme expression and drug metabolism in the placenta was not accounted for in the models. Kinetic data of acetaminophen metabolites measured in the *ex vivo* cotyledon perfusion assay could be used to assess enzyme abundance in the placenta, although this is complicated by the apparent back-conversion of metabolites to the parent compound (20). Additionally, results of the parameter optimization suggested some identification difficulties when fitting the parameters. In fact, *f*_*in*_ and *f*_*out*_ showed a relative strong correlation with the partition coefficients indicating that either the permeability or the partitioning should be ideally fixed to avoid non-identification issues. Further studies are needed to better inform these parameter separately.

In conclusion, this study presents a novel *in silico* cotyledon perfusion model consisting of seven compartments that can be used to mechanistically investigate placental drug transfer. The structure of this model is congruent with that of the placental compartments in the maternal-fetal PBPK model which allows a direct transfer of parameters from the former in the latter model. While the time of delivery will determine the time of collection of paired samples, at least the maternal part of a study protocol can be informed by predictions based on PK models, including PBPK models. The simulated accumulation of acetaminophen in the trophoblasts of the presented model might be of concern as this could potentially affect prostaglandin synthesis and subsequently fetal exposure to prostaglandins. Yet, it should also be stressed again that the measured drug concentrations in the maternal and fetal reservoirs were only to a limited extent informative of accumulation in the trophoblasts, as indicated by the sensitivity analysis result. Further studies should investigate potential accumulation as well as placental metabolism of acetaminophen.

The developed *in silico* cotyledon perfusion model is freely shared on OSP GitHub (https://github.com/Open-Systems-Pharmacology) for further applications and/or refinements that were beyond the scope of this study. Importantly, due to the mechanistic nature of the developed models, predictions can, in principle, be scaled to earlier stages of pregnancy (at least to the early second trimester when the formation of the placental barrier is completed). As clinical data are difficult to obtain at earlier stages of pregnancy, PBPK modeling approaches may constitute a potentially powerful tool to evaluate and investigate placental drug transfer and ultimately improve pharmacotherapy in pregnant women.

## Data Availability Statement

The datasets analyzed for this study are referenced in the article. Clinical data reported by Mehraban et al. (18) can be obtained from Nisha Lakhi (NLakhi@rumcsi.or) upon reasonable request.

## Author Contributions

PM, KA, and AD designed the research and wrote the manuscript. PM and AD performed the research. PM, BN, JA, KC, KA, NL, and AD analyzed the data. All authors contributed to the article and approved the submitted version.

## Conflict of Interest

AD is an employee of Bayer AG and uses Open Systems Pharmacology software, tools, and models in his professional role. The remaining authors declare that the research was conducted in the absence of any commercial or financial relationships that could be construed as a potential conflict of interest.

## Publisher's Note

All claims expressed in this article are solely those of the authors and do not necessarily represent those of their affiliated organizations, or those of the publisher, the editors and the reviewers. Any product that may be evaluated in this article, or claim that may be made by its manufacturer, is not guaranteed or endorsed by the publisher.
